# The Cancer/Testis Antigen Gene *VCX2* Is Rarely Expressed in Malignancies but Can Be Epigenetically Activated Using DNA Methyltransferase and Histone Deacetylase Inhibitors

**DOI:** 10.3389/fonc.2020.584024

**Published:** 2021-02-09

**Authors:** Mie K. Jakobsen, Sofie Traynor, Mette Stæhr, Pascal G. Duijf, Aaraby Y. Nielsen, Mikkel G. Terp, Christina B. Pedersen, Per Guldberg, Henrik J. Ditzel, Morten F. Gjerstorff

**Affiliations:** ^1^ Department of Cancer and Inflammation Research, Institute for Molecular Medicine, University of Southern Denmark, Odense, Denmark; ^2^ Institute of Health and Biomedical Innovation, Faculty of Health, School of Biomedical Sciences, Queensland University of Technology, Brisbane, QLD, Australia; ^3^ University of Queensland Diamantina Institute, Translational Research Institute, The University of Queensland, Brisbane, QLD, Australia; ^4^ Molecular Diagnostics Group, Danish Cancer Society Research Center, Copenhagen, Denmark; ^5^ Department of Oncology, Odense University Hospital, Odense, Denmark; ^6^ Academy of Geriatric Cancer Research (AgeCare), Odense University Hospital, Odense, Denmark

**Keywords:** VCX2, cancer/testis (CT) antigen, Immunotherapy, DNA methyl transferase (DNMT) inhibition, Histone deacetylase inhibitors

## Abstract

Identification of novel tumor-specific targets is important for the future development of immunotherapeutic strategies using genetically engineered T cells or vaccines. In this study, we characterized the expression of VCX2, a member of the VCX/Y cancer/testis antigen family, in a large panel of normal tissues and tumors from multiple cancer types using immunohistochemical staining and RNA expression data. In normal tissues, VCX2 was detected in the germ cells of the testis at all stages of maturation but not in any somatic tissues. Among malignancies, VCX2 was only found in tumors of a small subset of melanoma patients and thus rarely expressed compared to other cancer/testis antigens such as GAGE and MAGE-A. The expression of *VCX2* correlated with that of other VCX/Y genes. Importantly, we found that expression of VCX2 was inversely correlated with promoter methylation and could be activated by treatment with a DNA methyltransferase inhibitor in multiple breast cancer and melanoma cell lines and a breast cancer patient-derived xenograft. The effect could be further potentiated by combining the DNA methyltransferase inhibitor with a histone deacetylase inhibitor. Our results show that the expression of VCX2 can be epigenetically induced in cancer cells and therefore could be an attractive target for immunotherapy of cancer.

## Introduction

Cancer immunotherapy can provide clinical benefit and is the only known treatment with curative potential in the advanced setting, but identification of targets for safe and effective intervention remains challenging. During carcinogenesis the cell protein repertoire changes, resulting in expression of tumor antigens. Among these, cancer/testis (CT) antigens are highly promising targets for immunotherapy, due to their restricted expression pattern and immunogenic nature (1). Expression of CT antigens are limited to germ cells of the testis among healthy adult tissues, but aberrant expression is observed in cancers of multiple histological origin (2–4). Because testis is an immune-privileged organ, CT antigens can be considered neoantigens when expressed in tumors and elicit an immune response that is highly cancer specific. In agreement with this, trials with vaccines and adoptive T-cell therapies targeting CT antigens have shown promising results (5–10). However, effective responses are restricted by the heterogeneous expression pattern characterizing CT antigens (11, 12). CT antigen expression is limited to a subset of patients with a specific tumor type and often only a subset of cancer cells within tumors exhibit CT antigen expression. Therefore, strategies targeting multiple CT antigens may be required for achieving effective responses in a broad range of patients and characterization of the expression patterns of additional CT antigens can provide new highly cancer-specific targets for immunotherapeutic treatment of different cancers.

DNA methylation of promotor regions is a main regulator of expression of multiple CT antigen genes, where low methylation is correlated with an increase in gene expression. Therefore, expression of multiple CT antigens is upregulated in some cancer types and tumors due to a carcinogenesis-related genome-wide hypomethylation and, importantly, the expression can be induced or enhanced in CT antigen-negative tumors/cancer cells by treatment with DNA methyltransferase inhibitors. Consequently, DNA methyltransferase inhibitors have the potential to induce the expression of a broad range of CT antigens and improve the outcome of immunotherapy (13–16). Thus, cancer immunotherapy may be supplemented with DNA methyltransferase inhibitors to enhance clinical efficacy.

Members of the variable charge, X-linked/Y-linked (VCX/Y) gene family are potential new targets for immunotherapy. The VCX/Y genes encode a group of small, positively charged proteins, which are expressed exclusively in male germ cells among normal tissues (17, 18). The members are *VCX*, *VCX2*, *VCX3A*, *VCX3B*, *VCY*, and *VCY1B*. The X-linked gene members are clustered together on a region of chromosome Xp22. They share a highly homologues N-terminal region (>98% identity) but differ in their C-terminal region by different copy numbers of a 10 amino acid repeat segment (19). The function of the proteins remains largely unknown, but studies show that the proteins are located in the nucleus (18). Members of the VCX/Y family have recently been found to be potential immunotherapeutic targets for non-small cell lung cancer (NSCLC) (20, 21) and antibodies directed against the VCX protein were found in these patients, indicating that VCX is immunogenic (22). Furthermore, expression of VCX family genes is regulated by DNA methylation in lung cancer and colorectal cancer (20, 23). We aimed to further investigate the potential of VCX/Y as new targets for immunotherapy, focusing mainly on VCX2, by investigating VCX2 expression in normal and cancer tissues and examining the inducibility of *VCX2* by epigenetic treatment. We used a second-generation methyltransferase inhibitor, guadecitabine (SGI-110), which shows improved *in vivo* stability compared to the Food and Drug Administration (FDA)- and European Medicines Agency (EMA)-approved inhibitors, azacitidine and decitabine (24–26). VCX2 protein expression was investigated in multiple cancer types, but the focus was mostly on melanoma, as CT antigen expression tends to be high in this cancer type (27), and breast cancer, where new immunogenic targets are much needed.

## Materials and Methods

### Cell Culture

All cell lines were obtained from American Type Culture Collection (ATCC) and cultured in Dulbecco’s modified Eagle’s medium (DMEM, Sigma-Aldrich) or Roswell Park Memorial Institute (RPMI) medium supplemented with 10% fetal bovine serum (FBS, Sigma-Aldrich) and 1% penicillin-streptomycin. The cells were kept at 37°C and 5% CO2. All cell lines were routinely tested for the presence of mycoplasma with MycoAlert (Lonza). When harvested, the cells were washed with phosphate buffered saline (PBS, Sigma-Aldrich) and incubated with trypsin-EDTA (Sigma-Aldrich) for 5 min at 37°C. For immunohistochemical analysis, cells were treated with indicated concentrations of guadecitabine for 96 h and supplemented with 1 mM of valproic acid (Sigma-Aldrich) for 24 h where indicated.

### TCGA Data

Expression levels of genes encoding members of the VCX/Y family proteins were examined in breast cancer and melanoma using level 3 normalized, log_2_-transformed RNA sequencing data (Illumina HiSeq RNA Seq V2) from The Cancer Genome Atlas (TCGA) repository.

### Promoter Methylation Analyses of Tumors

Analysis of promoter methylation levels for indicated genes was performed as previously described (28) using the TCGA breast cancer (BRCA) and skin cutaneous melanoma (SKCM) cancer datasets. The β values for all CpG probes in the region from 1,500 bp upstream of the transcription start site (TSS) up until the end of the 5’UTR or the end of exon 1 (whichever is most downstream of the TSS) were used. For *VCY*, there were no probes in this region. For all other genes, there were no probes located between positions −1,500 bp and −200 bp with respect to the TSS. Altogether, the following probes were assessed: *VCX* [cg09018040, cg12528504 (each located in the region 200-1bp upstream of the TSS), cg23445828 (5’UTR)], *VCX2* [cg07380282 (5’UTR in exon 1)], *VCX3A* [cg04323915 (5’UTR in exon 1), cg13510648 (5’UTR)], *VCX3B* [cg07898500 (5’UTR in exon 1), cg23773680 (5’UTR)]. Per gene promoter, β values of all probes were averaged where indicated. Methylation levels were compared to expression levels from the respective TCGA Illumina HiSeq RNA Seq V2 (RSEM) BRCA and SKCM datasets. For statistical analyses, Mann-Whitney *U* tests were used.

### Targeted Methylation Analysis of Cell Lines

The methylation status of two regions of the *VCX2* promoter was determined by methylation-specific melting curve analysis (29). Five hundred nanograms of DNA were bisulfite converted (EZ DNA Methylation-Gold™ Kit; Zymo Research) and amplified using the LightCycler 2.0 system and the LightCycler FastStart DNA MasterPLUS SYBR Green I Kit (Roche) with the following primers: 5’-TTTTGAGGAATTTAGTTTGAT-3’ and 5’-AATCAAAATCAACAAACACA-3’ for region 1 (80 bp, 3 CpG sites), and 5’-AGGTGTGGAGGAATAGAATGTA-3’ and 5’-TATCACCCCTCCTAAACTCC-3’ for region 2 (121 bp, 4 CpG sites). Reactions were run in a total volume of 20 µl, including a final concentration of 1 mM each primer and 1 µl of bisulfite-converted DNA. PCR conditions were: 95°C for 10 min and 35 temperature cycles [95°C for 10 s, 60°C (region 1) or 62°C (region 2) for 20 s, 72°C for 30 s], followed by melting of the amplicon by increasing the temperature from 65 to 95°C. Fluorescence data was converted into melting peaks using the LightCycler software. The positive control for methylation was enzymatically methylated DNA (CpGenome Universal Methylated DNA; Sigma-Aldrich); the negative control for methylation was unmethylated DNA prepared by whole genome amplification (WGA; GenomePlex^®^, Sigma-Aldrich) of PBMC DNA.

### RNA Sequencing

MDA-MB-231 cells were cultured for 96 h with 1 µM guadecitabine and then left untreated for another 24 h before the cells were harvested in guanidium thiocyanate. Purified RNA from three independent experiments was prepared for sequencing using the TruSeq RNA sample preparation kit according to manufacturer’s instructions (Illumina) and sequenced using the Illumina HiSeq 1500 system. RNA sequencing reads were aligned to the human genome (hg19) using the Spliced Transcripts Alignment to a Reference (STAR) software (30) with default parameters. Tags in exons were counted using iRNA-seq (31). All samples were normalized to library size and presented as reads per Kb.

### Quantitative RT-PCR

RNA was purified from cells using RiboZol (VWR) followed by cDNA synthesis performed with the RevertAid Premium Reverse Transcriptase Kit from Fermentas. Quantitative real-time PCR was performed using SYBR green based expression analysis (Applied Biosystems) in combination with QuantiTect primers: VCX (QT01018647), VCX2 (QT00035980), VCX3A (QT00043526), VCX3B (QT01036196), VCY (QT00210126), VCY1B (QT00213374).

### Transfection of Cell Lines

HEK293 or A375 melanoma cells were transfected with VCX (NM_013452) and VCX2 (NM_016378) pCMV-entry expression plasmids for expression with C-terminal Myc-DDK-tag (OriGene) using Optifect (Life Technologies) according to the manufacturer’s recommendations.

### Western Blotting

Cells were lysed in RIPA buffer, resolved by 4-20% SDS-PAGE and electroblotted onto a PVDF membrane. The membrane was blocked in PBS, 0.1% Tween-20, 5% non-fat dry milk powder and incubated with anti-VCX2 (1:1000; ab188344; Abcam) or anti-FLAG (1:1000; M2; Sigma-Aldrich). The blot was further stained with horseradish peroxidase-conjugated goat anti-mouse or rabbit IgG (DakoCytomation Denmark A/S, Glostrup, Denmark) and developed with an ECL Western Blot kit (Amersham Biosciences, Hilleroed, Denmark, 1:100.000). All antibody incubation and washing steps were carried out in PBS, 0.1% Tween-20.

### Immunohistochemistry

Tissues sections were deparaffinized and treated with 1.5% H_2_O_2_ in Tris-buffered saline (pH 7.5) for 10 min to block peroxidase activity. Samples were then washed in TNT buffer (0.1 m Tris, 0.15 m NaCl, 0.05% Tween-20, pH 7.5) and subjected to different antigen retrieval protocols, including microwave boiling for 15 min in (1) T-EG buffer (10 mm Tris, 0.5 mm EGTA, pH 9.0), (2) 10 mm citrate buffer (pH 6.0), or (3) Dako Target Retrieval Solution (Dako S1699), or proteolytic treatment using (4) 0.05% protease type XIV (pronase E, Sigma, cat. no. P5147) in TBS (pH 7.0) for 15 min at 37°C. Optimal antigen retrieval was achieved with microwave boiling in T-EG buffer for 15 min. Sections were then incubated with rabbit anti-VCX2 (ab188344; Abcam) diluted in antibody diluent (S2022, DAKO Cytomation, Glostrup, Denmark) for 1 h at room temperature. Then, samples were washed with TNT, incubated with EnVision Flex/HRP+ for 30 min, washed again, and incubated with 3,3′-diaminobenzidine (DAB)+ substrate-chromogen for 10 min. Finally, samples were washed again with H_2_O and counterstained with Mayers hematoxylin before mounting in AQUATEX (Merck Inc., Whitehouse Station, NJ, USA). Normal tissues analyzed for VCX2 expression were: testis, placenta, cerebellum, tonsil, glandula submandibularis, thymus, thyroid gland, spleen, esophagus, ventricle, duodenum, colon, gall bladder, liver, pancreas, kidney, bladder, prostate, corpus uterus, cervix uterus, lung, skin, and skeletal muscle.

### Immunofluorescent Staining

Cells grown on glass slides were fixed in 4% formaldehyde for 10 min, permeabilized in 0.2% Triton X100, PBS for 5 min and treated with 3% BSA, PBS for 30 min. Immunostaining was done with anti-DDK/FLAG (Clone M2, Sigma-Aldrich) in 1% BSA, PBS, and goat anti-mouse IgG (H+L) cross-adsorbed Alexa Fluor 488 (Thermo Fisher Scientific). Cells were mounted under cover slides with ProLong Gold Antifade with DAPI (Life Technologies) and imaging was performed with an Olympus IX73 microscope fitted with a PlanApo N 60X/1.42 oil objective.

### Patient Derived Xenograft Experiments

Female NOG CIEA mice (NOD.Cg-Prkdc^SCID^ ll2rg^tm1Sug^/JicTac, Tatonic) were used for all *in vivo *experiments. Tissues from from female triple-negative breast cancer (TNBC) patients undergoing routine treatment at Odense University Hospital were used to generate the patient derived xenograft (PDX) tumors. The tissue specimens were embedded into extracellular matrix (ECM, Sigma-Aldrich) which had already been injected into the mammary fat pad of anesthetized mice. All PDXs were kept at low passages (<5). The mice were randomized to different treatment groups when macroscopic or palpable tumors were formed. Mice were treated with 24.4 mg/kg guadecitabine subcutaneously injected in the neck skinfold. The control group received vehicle (guadecitabine diluent buffer). All animal experiments were approved by the Experimental Animal Committee of The Danish Ministry of Justice and were performed at the animal core facility at the University of Southern Denmark. Animals were euthanized if they showed any adverse signs of disease, including weight loss, paralysis, or general discomfort. Mice were housed under pathogen-free conditions with *ad libitum* food and water. Tumors and organs were removed and fixed in formalin for 48 h and embedded in paraffin.

## Results

### VCX2 Is a Chromatin-Associated Protein Expressed in a Small Subset of Tumors

**Figure 1:**
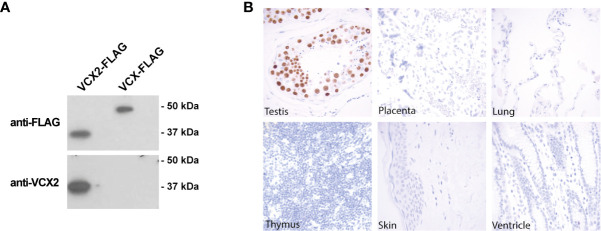
VCX2 protein expression is limited to testis among healthy adult tissues. **(A)** The specificity of a VCX2 specific antibody used for immunohistochemical analysis toward VCX2 was confirmed by western blotting using lysates from HEK293T cells with ectopic expression of VCX2 and VCX. The antibody only recognized VCX2 and not VCX or other endogenously expressed proteins. **(B)** VCX2 protein expression was investigated in a panel of 23 different normal adult tissues. Expression was only observed in testis among these tissues, where nuclear staining was seen in germ cells at all stages of spermatogenesis. Only a subset of tissues is shown. Magnification: × 40.

To investigate the potential of VCX2 as a novel therapeutic target for immunotherapy, we analyzed its expression in normal tissues and different types of cancer using immunohistochemical staining. The specificity of an antibody raised against a unique structure in VCX2 (amino acids 118–139) was first validated by western blotting using HEK293 cells with ectopic expression of VCX2 and its highly identical homologue VCX. The antibody was demonstrated to recognize VCX2 but not VCX or any protein endogenously expressed in HEK293 cells (**Figure 1A**), suggesting that it was highly specific for VCX2.

Next, we evaluated the expression of VCX2 in a panel of 23 different normal tissues using immunohistochemical staining with this antibody. VCX2 was only expressed in testis among these tissues, where strong nuclear staining was seen in germ cells of all stages of spermatogenesis (**Figure 1B**). The staining pattern in testis resembled that of VCX, which however seemed to be expressed at higher levels in spermatogonia compared to cells at more mature stages of spermatogenesis (21). No expression of VCX2 was seen in the placenta or the cerebellum, where other CT antigens have been identified (32). The fact that VCX2 expression is restricted to testis, which is an immune-privileged organ, diminishes the risk of off-tumor toxicity when targeting VCX2 and increases the potential of VCX2 as a target for immunotherapy.

**Figure 2:**
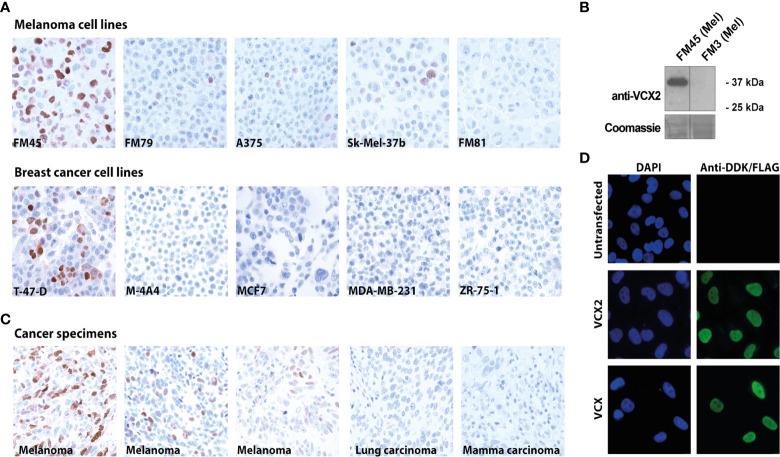
VCX2 expression in cancer and subcellular localization. **(A)** VCX2 protein expression was observed in 4/19 melanoma cell lines (FM45, FM79, A375, and Sk-Mel-37b) and 1/11 breast cancer cell lines (T-47-D) (see **Supplementary Table 1** for a complete list). **(B)** The antibody was confirmed to recognize a protein similar in size to VCX2 in melanoma cell lysate from cells positive in immunohistochemical staining (FM45) and did not react with a lysate from cells negative in immunohistochemical staining (FM3). **(C)** Among 261 cancer specimens derived from 19 different histological origins, VCX2 protein expression was only observed in melanoma, where expression was seen in 4/31 cancer specimens (see **Table 1** for a complete list). Only a subset of cancer cell lines and cancer specimens are shown. **(D)** Investigation of VCX2 subcellular localization in A375 cells, using immunocytochemistry, showed the presence of the protein only in the nucleus, with a localization pattern similar to chromatin. Magnification: × 40 **(A, C)** × 60 **(D)**.

We then investigated VCX2 protein expression in a large panel of cell lines from breast cancer and melanoma (**Supplementary Table 1**). The VCX2 protein was expressed in only 1/11 breast cancer cell lines (i.e., T-47-D) and in 4/19 melanoma cell lines (i.e., FM45, FM79, A375, and Sk-Mel-37b) and three of the cell lines showed a very low number (<5%) of VCX2 positive cells (i.e., FM79, A375, and Sk-Mel-37b) (**Figure 2A**). Using western blotting we confirmed that the antibody indeed recognized a band corresponding to VCX2 in melanoma cells (**Figure 2B**). In comparison, GAGE and MAGE-A CT antigens were expressed in 10/11 and 4/11 breast cancer cell lines and 6/19 and 16/19 melanoma cell lines, respectively (**Supplementary Data 1**). We also investigated VCX2 protein expression in 261 cancer specimens from multiple histological origins, summarized in **Table 1**. Expression was only observed in melanoma, where VCX2 protein was detected in 4/31 specimens (**Figure 2C**). Again, VCX2 was much less frequently expressed in cancers compared to GAGE and MAGE-A CT antigens, which we previously detected in 10-40% of tumors of multiple cancer types (e.g., melanoma, lung cancer, bladder cancer, and breast cancer) (33).

**Table 1:** The expression of VCX2 in different types of malignancies as determined by immunohistochemical staining.

Tumor type	VCX2 expression
	
Appendix carcinoid	0/5
Bladder carcinoma	0/10
Breast carcinoma	0/35
Cervix carcinoma	0/8
Colorectal carcinoma	0/2
Endometrial carcinoma	0/15
Gallbladder carcinoma	0/2
Gastric carcinoma	0/9
Kidney carcinoma	0/12
Lung carcinoma total	0/54
Adenocarcinoma	0/14
Large cell carcinoma	0/9
Small cell carcinoma	0/2
Planocellular carcinoma	0/29
Malignant melanoma Mesothelioma	4/31 0/5
Ovary carcinoma	0/9
Pancreatic carcinoma	0/2
Pheochromocytoma	0/3
Prostate carcinoma	0/8
Salivary gland carcinoma	0/5
Small intestine carcinoma	0/4
Thyroid carcinoma	0/10

Our analysis of VCX2 expression demonstrated that VCX2 was predominantly located in the nuclei of cells in both testis germ cells and melanoma. In accordance, a former study also indicated that members of the VCX/Y family are nuclear proteins (18). Furthermore, the VCX localization pattern in testis germ cells indicated that VCX2 was associated with chromatin in these cells. To investigate if VCX2 exhibited a similar nuclear distribution in cancer cells, we performed a detailed analysis of its nuclear localization in A375 melanoma cells using fluorescence microscopy (**Figure 2D**). This showed that VCX2 is not homogeneously dispersed within nuclei but rather exhibits a localization pattern similar to DAPI (i.e., chromatin), suggesting that this protein is a chromatin-associated factor. A similar pattern was observed for VCX (**Figure 2D**).

**Figure 3:**
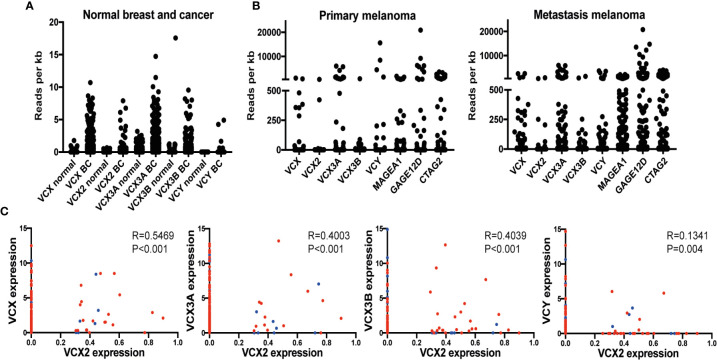
VCX is expressed in a subset of breast cancer and melanoma tumors and correlates with the expression of other VCX family members. **(A)** RNA expression of VCX family members in normal breast and breast cancer tissues. Data were generated using RNA sequencing data from the TCGA repository. Results are presented as relative expression levels (normalized number of reads). **(B)** Expression of VCX family members and GAGE, MAGE-A1, and CTAG1B (NY-ESO-1) CT antigens primary and metastatic melanoma tumors. Data were generated using RNA sequencing data from the TCGA repository. Results are presented as relative expression levels (normalized number of reads). **(C)** Spearman correlation between expression of VCX2 and other members of the VCX family in primary (blue) and metastatic (red) melanoma tumors. VCX significantly correlated with all other VCX genes. The p-values were calculated using the (unpaired) Mann-Whitney *U* test.

To support the results of our immunohistochemical analysis of VCX2 expression we analyzed the expression of the *VCX2* gene in normal and cancerous breast and skin tissue using RNA sequencing data from the TCGA repository (**Figures 3A, B**). We further compared the expression of *VCX2* to that of other *VCX/Y* gene members among normal and cancer tissues. As expected, all investigated VCX/Y genes (*VCX, VCX2, VCX3A, VCX3B*, and *VCY*) showed no/very low expression in normal breast tissue. *VCX/Y* gene expression in normal skin was difficult to elucidate from these data due to a very low number of samples (n=1) in the TCGA repository. In general, *VCX/Y* genes were not significantly upregulated in breast cancer and melanoma lesions, but a subset of patient tumors exhibited highly increased expression compared to normal tissue or the baseline of tumor samples. VCX2 was expressed in only 2/103 primary melanomas and 4/368 metastatic melanomas, and only expressed at a very low level in a subset of breast cancers. This supported the result of our immunohistochemical analysis of VCX2 expression and further indicated that this CT antigen is a potential target in only a small subset of melanomas. Other VCX/Y genes were expressed at higher frequencies in melanoma, but also expressed at very low levels in breast cancers. The expression of VCX2 was significantly correlated to that of other members of the VCX/Y family (**Figure 3C**), suggesting that regulation of the genes is guided by similar mechanisms and that they do not supplement each other as targets of immunotherapy. Our results suggest that VCX2 may be a target for immunotherapy in only a small subset of melanoma patients. However, as expression of multiple CT antigens is shown to be upregulated by epigenetic treatment, we wanted to investigate if that is also true for VCX2.

### VCX2 Protein Expression May Be Upregulated by DNA Methyltransferase Inhibitors in Cancer to Enable Targeting

**Figure 4:**
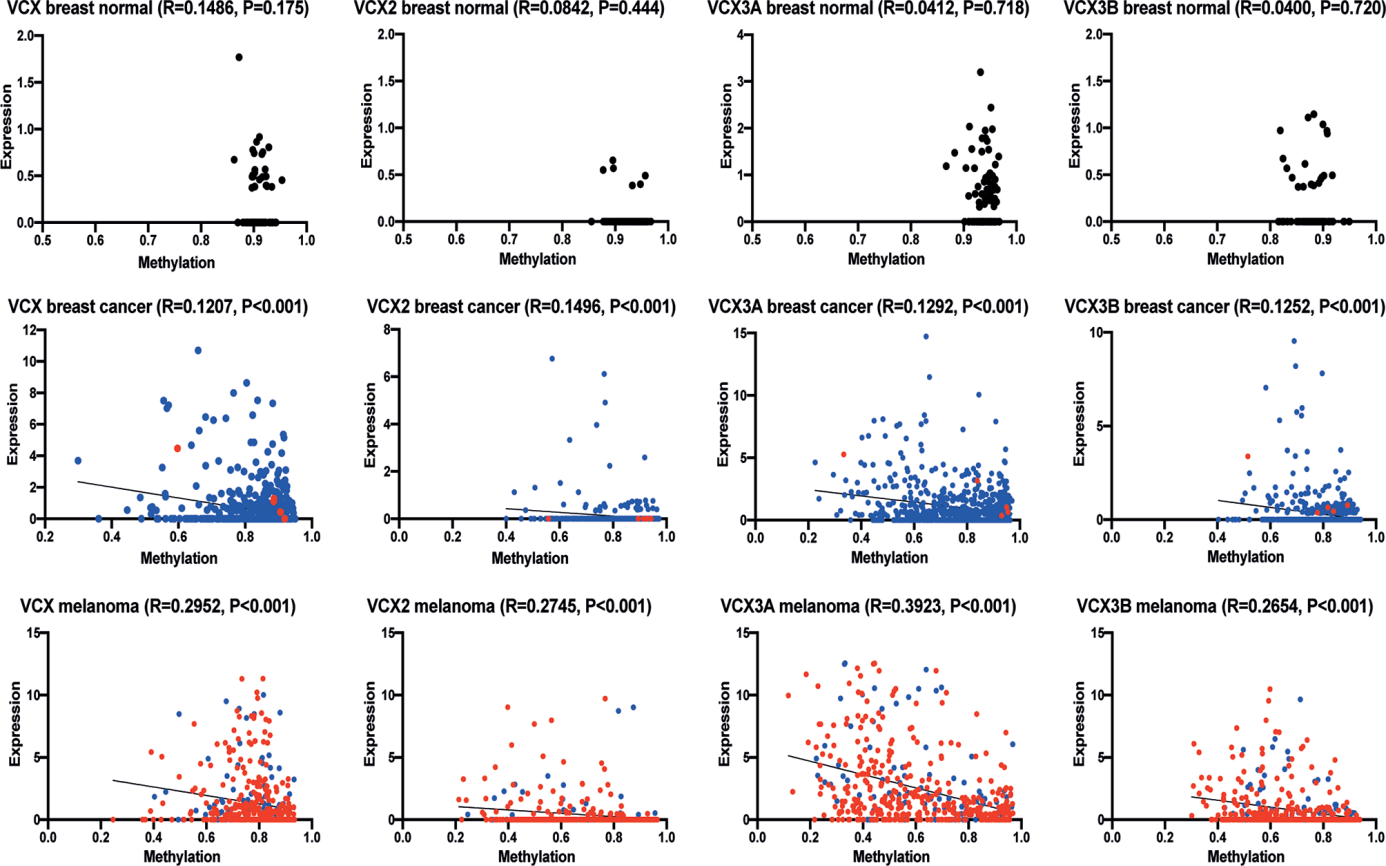
Correlation of VCX gene promotor methylation levels and mRNA expression. Spearman correlation between average promotor methylation levels, shown as ß-values and mRNA expression of all VCX family genes in healthy and cancerous breast tissue and melanoma. A significant correlation was observed for all VCX family genes in both breast cancer and melanoma, but not in healthy breast tissue. Blue indicates primary tumor specimens and red indicates metastatic tumor specimens. The p-values were calculated using the (unpaired) Mann-Whitney *U* test.

CT antigens are often regulated by CpG methylation of promotor regions, where decreased methylation is correlated with an increase in gene expression. With the potential of clarifying if the VCX/Y family genes could be induced by demethylation, we investigated if the expression of *VCX2* and other *VCX* genes were correlated with promoter methylation in breast cancer and melanoma (**Figure 4A**). Gene expression significantly and inversely correlated with methylation for all VCX family members in both breast cancer and melanoma. These results suggest that VCX family gene expression is dependent on CpG methylation in breast cancer and melanoma.

**Figure 5:**
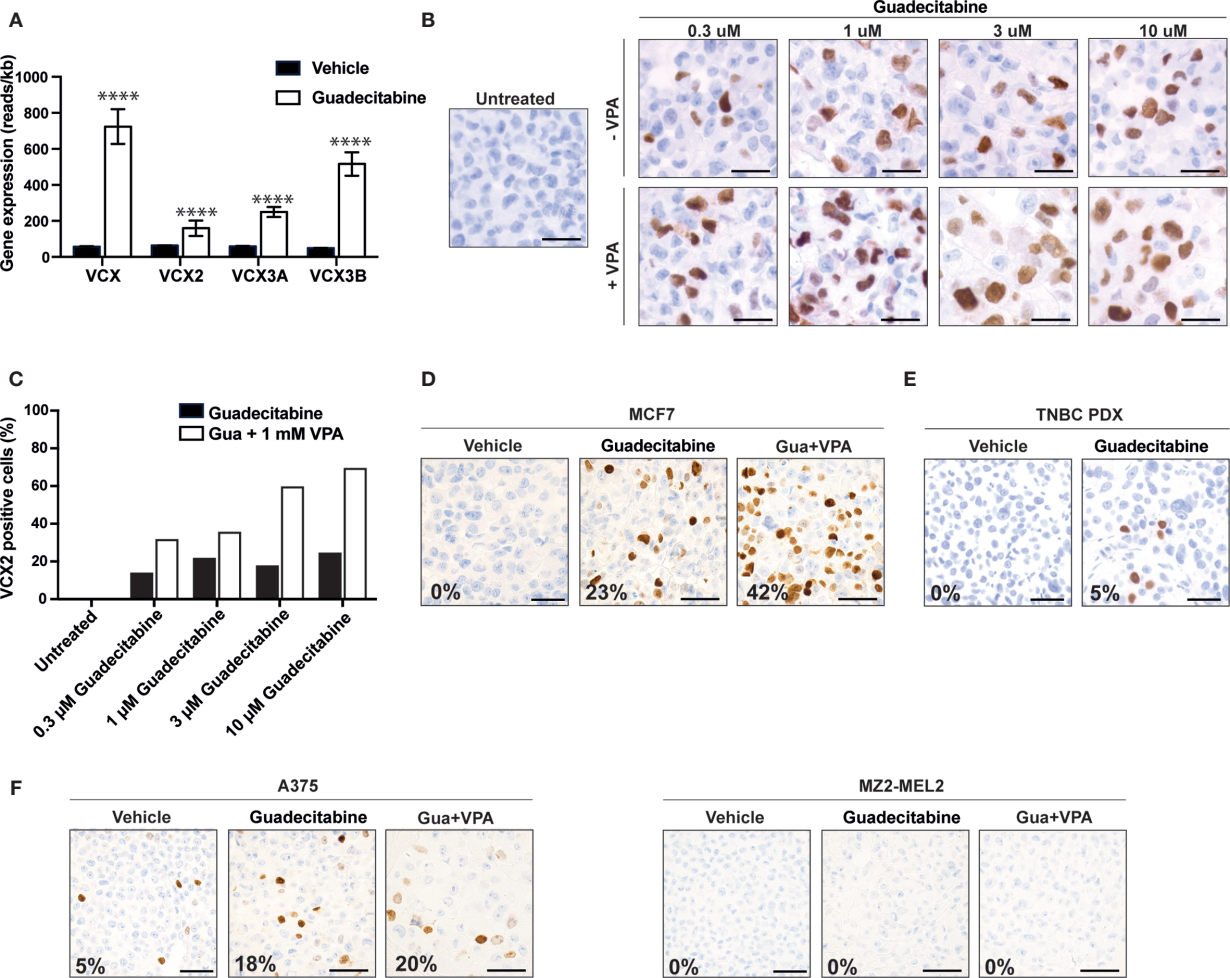
Upregulation of VCX2 expression by epigenetic treatment in breast cancer and melanoma cells. **(A)** VCX family gene expression was investigated by RNA sequencing in MDA-MB-231 breast cancer cells after treatment with either vehicle (black bars) or guadecitabine (white bars) for 96 h. All VCX genes were significantly upregulated by treatment with guadecitabine. The experiment was performed with three biological replicates. **(B)** The effect of combining DNA methyltransferase inhibitors and histone deacetylase inhibitors were investigated using guadecitabine and valproic acid. MDA-MB-231 cells were treated with increasing concentrations of guadecitabine (0.3 to 10 μM) with or without 1 mM valproic acid for 96 h and VCX2 protein expression was subsequently evaluated using immunocytochemical staining. The experiment was performed with two biological replicates. A representative replicate is shown. **(C)** Quantification of cells from **(B)**. **(D)** MCF7 breast cancer cells were treated with 0.5 mM of guadecitabine with or without 1 mM valproic acid (VPA) for 96 h and VCX2 protein expression was subsequently evaluated using immunocytochemical staining. The experiment was performed with two biological replicates. A representative replicate is shown. Approximate frequency of positive cells is shown in bottom left corner. **(E)** PDX tumors from a metastatic TNBC patient were established in NOG mice and when tumors reached a size of 2–3 mm, the mice were randomized into treatment groups based on tumor size. The mice were treated with either 24.4 mg/kg guadecitabine or vehicle every fifth day for a total of four times and VCX2 expression was subsequently evaluated by immunohistochemistry. The experiment was performed with three biological replicates. A representative replicate is shown. Approximate frequency of positive cells is shown in bottom left corner. Scale bars = 50 µM. **(F)** A375 and MZ2-MEL melanoma cells were treated with 0.5 mM of guadecitabine (gua) with or without 1 mM valproic acid (VPA) for 96 h and VCX2 protein expression was subsequently evaluated using immunocytochemical staining. Representative pictures are shown. Approximate frequency of positive cells is shown in bottom left corner. Scale bars = 50 µM.

Because we found a correlation between CpG methylation and VCX family gene expression and because earlier studies have indicated that some VCX/Y genes are regulated by DNA methylation, we investigated if the expression of members of the VCX gene family could be up-regulated in cancer cells by treatment with the DNA methyltransferase inhibitor guadecitabine. First, we investigated the expression of VCX genes in MDA-MB-231 triple-negative breast cancer cells after treatment with either vehicle or guadecitabine using RNA sequencing (**Figure 5A**). Baseline expression of all VCX family genes was low in this cell line, but they were all significantly upregulated by guadecitabine. We then investigated if guadecitabine also upregulated VCX2 on protein level. MDA-MB-231 breast cancer cells were treated with increasing amounts of guadecitabine (0.3 μM – 10 μM) for 4 days and subjected to immunohistochemical staining (**Figures 5B, C**). This showed that VCX2 was induced in up to 24% of the cells upon guadecitabine treatment, with the maximal effect achieved already with 1 µM. Next, we investigated whether the histone deacetylase inhibitor valproic acid in combination with guadecitabine could enhance VCX2 expression in MDA-MB-231 cells (**Figures 5B, C**). The number of VCX2-positive cells was considerably increased using the combination compared to guadecitabine alone, with 60 and 70% of cells being positive when treated with valproic acid in combination with 3 and 10 µM guadecitabine, respectively. A similar pattern was seen with the hormone receptor positive breast cancer cells MCF7; guadecitabine increased the frequency of VCX2-positive cells (from 0 to 23%) and again this was potentiated by addition of valproic acid (42%) (**Figure 5D**).

We further investigated the level of VCX2 expression in a clinically relevant PDX mouse model established from a metastatic lesion from a TNBC patient (TNBC-9228) upon guadecitabine treatment. TNBC PDX tumors were established in NOG mice and when tumors reached a size of 2-3 mm, the mice were randomized into treatment groups based on tumor size. The mice were treated with either 24.4 mg/kg guadecitabine or vehicle every fifth day for a total of four times, as this dosing strategy was effective for induction of MAGE and NY-ESO-1 CT antigens in *in vivo* models of leukemia and ovarian cancer (34, 35). VCX2 expression in PDX tumors was subsequently evaluated by immunohistochemistry (**Figure 5E**). No VCX2 expression was observed in vehicle-treated PDX tumors, but VCX2 expression was observed in a small number of cells (<5%) in the guadecitabine-treated PDX tumor. Since our *in vitro* results suggested that the combination of guadecitabine and valproic acid was beneficial for enhancement of VCX2 expression, we wanted to test the effect of this strategy on TNBC PDX tumors as well. However, the combination of guadecitabine and valproic acid resulted in premature death in NOG mice and the experiment was not completed.

Next, we investigated if epigenetic priming could also induce VCX2 in melanoma cells (**Figure 5F**). About 5% of A375 cells were already positive for VCX2 and guadecitabine-treatment enhanced this to 18%, but the frequency of positive cells was not significantly increased (20%) with the combination of guadecitabine and valproic acid. Epigenetic treatment of MZ2-MEL melanoma cells with guadecitabine and valproic acid did not produce any VCX2-positive cells (**Figure 5G**).

These results show that VCX2 protein expression can be upregulated by epigenetic inhibitors in breast cancer and melanoma cells. However, not all tumor cells respond to the treatment.

### VCX2 Expression in Breast Cancer Cells Is Associated With Loss of Promoter Methylation

**Figure 6:**
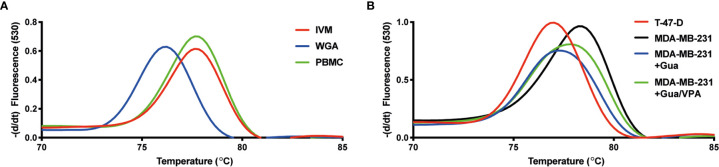
VCX2 expression in breast cancer cells is associated with loss of promoter methylation. The level of *VCX2* promoter methylation in peripheral blood mononuclear cells (PBMCs) and breast cancer cells lines was investigated using bisulfite conversion followed by PCR melting point analysis. **(A)** The methylation level in PBMCs was similar to that of *in vitro* methylated DNA (IVM) and distinct from that of unmethylated DNA prepared by whole genome amplification (WGA). **(B)** The analysis was performed on cells with (T-47-D) or without (MDA-MB-231) endogenous expression of VCX2 and on cells with guadecitabine/valproic acid-induced expression of VCX2 [MDA-MB-231 + guadecitabine (Gua)/valproic acid (VPA)], which demonstrated inverse correlation between *VCX2* promoter methylation and gene expression. Two different promoter regions were analyzed with highly similar results (only data for region 2 are shown). The experiment was performed with two biological replicates.

To further investigate the importance of promoter DNA methylation for regulating the activity of *VCX2*, we investigated the CpG methylation levels of two different regions adjacent to the transcription start site using bisulfite conversion and PCR melting point analysis. Region 1 was located upstream from the transcription start site (−71 to −110 bp) and included three potential CpG methylation sites, whereas region 2 was located in the beginning of exon 1 (215 to 265 bp) and included four potential methylation sites. As expected, the *VCX2* promoter was highly methylated in peripheral blood mononuclear cells (PBMCs) similar to *in vitro* methylated control DNA (**Figure 6A**). This suggested that *VCX2* is silenced by hypermethylation in normal somatic tissues similarly to other CT antigen genes. A high level of *VCX2* methylation was also observed in the VCX2-negative MDA-MB-231 cells. In contrast, the VCX2-positive T-47-D cells exhibited a *VCX2* promoter methylation level similar to unmethylated control DNA (**Figure 6B**). Furthermore, epigenetic induction of VCX2 expression in MDA-MB-231 cells with guadecitabine, alone or in combination with valproic acid, was associated with loss of *VCX2* promoter methylation (**Figure 6B**). The methylation patterns for regions 1 and 2 were highly similar. These data showed that the transcriptional activity of *VCX2* in breast cancer cell lines was inversely correlated with promoter methylation and suggest that promoter methylation is instrumental for the regulation of this gene similarly to other CT antigen genes.

## Discussion

The development of novel immunotherapies for treatment of solid cancers, such as adoptive T cell therapy and vaccines, is to some degree limited by the lack of suitable targets with high cancer specificity and broad tumor expression. Therefore, it is crucial to identify and characterize the expression pattern of potential targets in normal tissues and different types of cancer. CT antigens are promising targets for cancer immunotherapy due to their highly restricted expression pattern in normal tissues, but the extent of expression of different CT antigens in cancer is highly variable. It is therefore essential to perform detailed analysis of individual CT antigens to identify the best targets for immunotherapy (4, 36).

In this study, we used a novel antibody to characterize the expression pattern of VCX2 to evaluate its therapeutic potential. Although this protein was found to be strictly limited to testes germ cells in normal tissues and therefore could be a suitable target for immunotherapy, staining of a high number of tumor specimens from different types of cancer showed that VCX2 is only expressed in a small number of melanomas. This suggested that VCX2 is only a useful target for treatment of a subset of melanoma patients. RNA sequencing analysis also indicated that *VCX2* might be less frequently expressed in melanoma and breast cancer than other VCX gene family members (*VCX* and *VCX3A*). Furthermore, VCX members generally seemed to be expressed less frequently in tumors compared to well characterized CT antigens such as MAGE-A, GAGE, and NY-ESO-1.

Many CT antigen genes have been demonstrated to be inducible with DNA methyltransferase inhibitors and the combination of epigenetic modulation of tumor CT antigen expression and immunotherapy targeting CT antigens is an attractive approach for broadening the use of immunotherapy. Importantly, this strategy seems to be specific for cancer cells as induction of CT antigen expression was found in normal cells in multiple studies (1, 15, 16, 37–39). We found that the expression level of *VCX2* and other *VCX* genes correlate with promoter methylation in breast cancer and melanoma, suggesting that these genes can be induced by DNA methyltransferase inhibitors. Indeed, treatment of breast cancer and melanoma cell lines with the DNA methyltransferase inhibitor guadecitabine, alone or in combination with histone deacetylase (HDAC) inhibitor valproic acid, promoted loss of VCX2 promoter DNA methylation and induced *VCX2* expression. The epigenetic induction of *VCX2* expression was confirmed on the protein level and we further found that the width and amplitude of guadecitabine-induced VCX2 protein expression could be significantly increased by addition of the HDAC inhibitor in some samples. This suggested that combined DNA methyltransferase inhibitor and HDAC inhibition would be a better approach for enhancing VCX2 expression in clinical tumors. Although this strategy seems promising for enhancing antigen expression in tumors, the lack of effect in MZ2-MEL2 cells suggested that not all tumors will respond. Importantly, we also demonstrated that VCX2 could be detected in TNBC PDX tumors after guadecitabine treatment. The frequency of positive cells in PDX tumors were quite low, suggesting that the combination of DNA methyltransferase and HDAC inhibitors may be needed to achieve a clinically relevant response. Furthermore, targeting of VCX2 together with other CT antigens may increase the frequency of targetable cells within tumors to enhance the clinical benefit.

Several CT antigens have been demonstrated to confer hallmark phenotypes on cancer cells, including support of cell proliferation, repression of apoptosis, and enhancement of invasion/metastasis (1). Thus, the strategy of epigenetically inducing CT antigen expression in tumors may seem unattractive. However, epigenetic drugs like DNA methyltransferase and histone deacetylase inhibitors affect the expression of hundreds of genes, also including re-expression of tumor suppressor genes. Therefore, the net effect of treating tumor cells with these drugs has been well demonstrated to be reduced tumor growth and metastatic spread (40–43), and epigenetic enhancement of CT antigen expression seems to be a safe strategy for increasing tumor immunogenicity.

**Figure 7:**
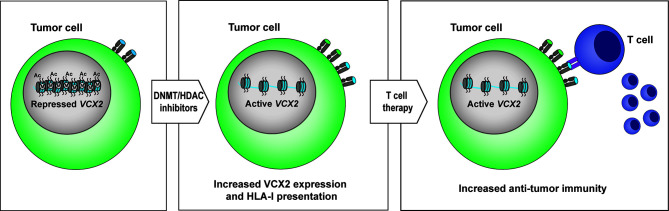
Proposed model for targeting VCX2 in tumors. *VCX2* is rarely expressed in tumors, but can be epigenetically activated by treatment with DNA methyltransferase (DNMT) and histone deacetylase (HDAC) inhibitors. Enhanced production of VCX2 will make tumor cells susceptible to anti-VCX2 T-cell responses or to T-cell therapy with T cells genetically modified to express VCX2-specific T-cell receptors.

Target selection is a major challenge to establishing long-lasting T-cell therapy of solid cancers due to the heterogeneity of antigen expression. Possible solutions include: 1) broadening the expression of antigens in tumors with epigenetic drugs, and/or 2) the use of polyvalent combinatorial antigen vaccines to increase the numbers of tumor cells targeted by the T-cell products. Although these strategies are highly feasible alone or in combination, further insight is needed into the expression patterns of individual tumor antigens to rationally design broadly reactive T-cell vaccines that maximize clinical efficacy. In this study, we have demonstrated that *VCX2* and other *VCX* genes are amenable to epigenetic induction by DNA methyltransferase and HDAC inhibitors in cancer cell lines and tumors and therefore may represent new targets for cancer immunotherapy (**Figure 7**). However, *in vitro* and *in vivo* studies with VCX2-directed T-cells are needed to validate the therapeutic value of epigenetically enhancing *VCX2* expression. To achieve this, relevant VCX2 T-cell epitopes will first have to be identified. Thus, further preclinical development is required before clinical strategies can be defined.

## Data Availability Statement

The original contributions presented in the study are publicly available. This data can be found here: https://www.ncbi.nlm.nih.gov/geo/, accession number GSE163468.

## Ethics Statement

The animal study was reviewed and approved by Experimental Animal Committee of The Danish Ministry of Justice.

## Author Contributions

MJ contributed to the design and experimental part of the study and writing of the manuscript. ST contributed to the experimental part of the study and writing of the manuscript. MS contributed to the experimental part of the study and writing of the manuscript. PD contributed to the design and experimental part of the study and writing of the manuscript. AN contributed to the design and experimental part of the study. MT contributed to the experimental part of the study. CP contributed to the experimental part of the study. HD contributed to the design of the study and writing of the manuscript. MG contributed to the design and experimental part of the study and writing of the manuscript. All authors contributed to the article and approved the submitted version.

## Funding

This work was supported by Pink Tribute, Læge Sofus Carl Emil Friis og Hustru Olga Doris Friis Legat, the Danish Cancer Society, the Academy of Geriatric Cancer Research (AgeCare), the Novo Nordisk Foundation and the Danish Research Council for Independent Research.

## Conflict of Interest

The authors declare that the research was conducted in the absence of any commercial or financial relationships that could be construed as a potential conflict of interest.
