# Supplementary material for: The Cancer/Testis Antigen Gene VCX2 Is Rarely Expressed in Malignancies but Can Be Epigenetically Activated Using DNA Methyltransferase and Histone Deacetylase Inhibitors
Source: Front Oncol. 2021 Feb 9;10:584024. doi: 10.3389/fonc.2020.584024 (PMC7900521; doi:10.3389/fonc.2020.584024)
Supplement: Supplementary file 1 [file Table_1.docx]

**Supplementary table 1. Expression of VCX, GAGE and MAGE-A cancer/testis antigens in breast cancer (BC) and melanoma (MEL) cell lines as determined by immunohistochemical staining**

| **Cell line** | **VCX** | **GAGE** | **MAGE-A** |
| --- | --- | --- | --- |
| BrCa-MZ-01 (BC) | - | + | + |
| BT-474 (BC) | - | - | - |
| CAL-51 (BC) | - | + | - |
| MCF7 (BC) | - | + | - |
| MDA-MB-157 (BC) | - | + | - |
| MDA-MB-231 (BC) | - | + | - |
| MDA-MB-435s (BC) | - | + | + |
| MDA-MB-468 (BC) | - | + | - |
| SKBR-3 (BC) | - | + | + |
| T-47-D (BC) | + | + | + |
| ZR-75-1 (BC) | - | + | - |
| A375 (MEL) | + | - | - |
| FM2 (MEL) | - | - | + |
| FM3 (MEL) | - | - | + |
| FM6 (MEL) | - | + | + |
| FM28 (MEL) | - | - | + |
| FM45 (MEL) | + | + | + |
| FM55-M1 (MEL) | - | - | - |
| FM55-P (MEL) | - | - | - |
| FM57 (MEL) | - | - | + |
| FM72 (MEL) | - | - | + |
| FM79 (MEL) | + | + | + |
| FM81 (MEL) | - | + | + |
| FM82 (MEL) | - | + | + |
| FM86 (MEL) | - | - | + |
| FM88 (MEL) | - | - | + |
| MZ-MEL-2 (MEL) | - | + | + |
| Sk-Mel-28 (MEL) | - | - | + |
| Sk-Mel-37b (MEL) | + | - | + |
| Sk-Mel-44 (MEL) | - | - | + |
